# Special Issue: Silicon Nanodevices

**DOI:** 10.3390/nano12121980

**Published:** 2022-06-09

**Authors:** Henry H. Radamson, Guilei Wang

**Affiliations:** 1 Research and Development Center of Optoelectronic Hybrid IC, Guangdong Greater Bay Area Institute of Integrated Circuit and System, Guangzhou 510535, China; 2 Beijing Superstring Academy of Memory Technology, Beijing 100176, China

In recent years, nanodevices have attracted a large amount of attention due to their low power consumption and fast operation in electronics and photonics, as well as their high sensitivity in sensor applications. For example, following Moore’s law and the technology roadmap, the structure of transistors has been scaled down by a constant factor in order to obtain lower power consumption. Today, the scaling down of CMOS technology is focusing more on low voltages and cost-effective processes to match the requirements of mobile phone chips [1].

As a result of CMOS evolution, a lot of integration difficulties have been overcome, enabling the architecture of CMOS technology to change from planar or 2D to 3D. During this technology development, many issues, e.g., contact resistance, defects and reliability, have arisen and have been solved, which could affect device performance [2,3]. As an approach at the end of the technology roadmap (3 nm node), Si channel material is being changed to SiGe or Ge material, and even III-V materials could be integrated in the future. This is due to their material properties, as they have significantly higher carrier mobilities of −40,000 cm^2^ V^−1^ s^−1^ for InGaAs (for electrons) and 1900 cm^2^ V^−1^ s^−1^ for Ge (for holes) compared to silicon, which has 1400 cm^2^ V^−1^ s^−1^ for electrons and 450 cm^2^ V^−1^ s^−1^ for holes [1]. In many cases, these are processed to be fin-like or nanowire transistors on insulator substrates. Special attention is paid to All Gate Around (GAA) transistors in vertical or horizontal directions. In such cases, GeSi/Ge multilayers are grown, and later, GeSi can be selectively etched to reach a nano-scale channel [4]. What is interesting is the choice of our transistor design beyond Moore’s era. Meanwhile, as a future type of computation that could be used when Moore’s Law ends, quantum computing is gaining considerable attention from academic and industrial communities. Group IV material devices will be an important aspect of quantum computing.

Currently, photonic and sensor devices have also attracted a large amount of interest. For example, materials are required for emissions in the infrared and terahertz range with high responsivity and low dark currents [5,6,7]. Therefore, many studies are seeking methods to decrease the defect density, in particular in device processing. The main goal is to find a monolithic solution where a material can be used for both photonic and electronic components on the chip. Ge and GeSn are materials that are recognized as excellent candidates as optoelectronic materials [8,9,10,11,12].

In this field, nanodevices are manufactured in 3D, and the emergence of electronics and photonics is inevitable. It is obvious that the complexity of our society requires technology to become more complicated in its design. In the future, electronic–photonic designs will be our ultimate goal in nanotechnology.

This Special Issue presents the fabrication and characterization of group IV nanostructures, nanodevices and nanosensors and their integration with photonics. The issue also covers optoelectronic materials and defect engineering as well as characterization.
